# Successful intravenous immunoglobulin therapy in paraneoplastic pemphigus associated with hepatocellular carcinoma^☆^

**DOI:** 10.1016/j.abd.2025.501153

**Published:** 2025-07-01

**Authors:** José Ramos, Cláudia Afonso, Ângela Roda, Cristina Fonseca

**Affiliations:** a Dermatology and Venereology Department, ULS Almada-Seixal, Almada, Portugal; b Gastroenterology Department, ULS Almada-Seixal, Almada, Portugal

*Dear Editor,*

Paraneoplastic pemphigus (PNP), or paraneoplastic multiorgan autoimmune syndrome (PAMS), is an autoimmune blistering disease characterized by severe, often intractable, mucous membrane involvement and polymorphous skin lesions, that is classically associated with hematological neoplasms and, more rarely, with solid tumors.^1^ Given the rarity of this disorder and the heterogeneity of clinical presentation, clinicians should maintain a high index of suspicion for PNP/PAMS to avoid delayed diagnosis. In 2021, the diagnostic criteria for PNP/PAMS were revised to better encompass the heterogeneous nature of this syndrome: the diagnosis is established when the three major criteria are met (mucositis with or without skin involvement, concomitant internal malignancy, and evidence of anti-plakin autoantibodies), or when two major and two minor criteria are present (acantholysis and/or interface lichenoid dermatitis observed in histopathology with or without keratinocyte necrosis; and direct IF showing intercellular and/or basement membrane staining).^2^ The treatment of PNP remains a clinical challenge.^3^ Systemic corticosteroids remain the first line of treatment for patients with PNP. Additional options for disease control include conventional steroid-sparing agents, rituximab, and intravenous immunoglobulin (IVIG).

Herein, we report a rare case of paraneoplastic pemphigus associated with hepatocellular carcinoma, successfully controlled with IVIG therapy in combination with systemic corticosteroids.

An 81-year-old Caucasian male, recently diagnosed with inoperable hepatocellular carcinoma was referred to our department for painful oral mucositis with six months' duration, associated with dysphonia, dysphagia, and significant weight loss (34%). He had already completed several therapies for oral mucositis, including antiviral, antifungal, and systemic corticosteroid therapy, without improvement. Also noteworthy, in the context of the dysphagia investigation, he had undergone an upper gastrointestinal endoscopy that revealed low-grade erosive esophagitis.

In addition to oral mucositis, in the past three months, the patient developed a disseminated skin eruption characterized by erythematous-brownish hyperkeratotic papules and plaques on the trunk (Fig. 1A and B). Clinical examination of the oral mucosa showed erosions on the tongue, buccal, and labial mucosa, covered by hemorrhagic crusts (Fig. 1C).

**Fig. 1:**
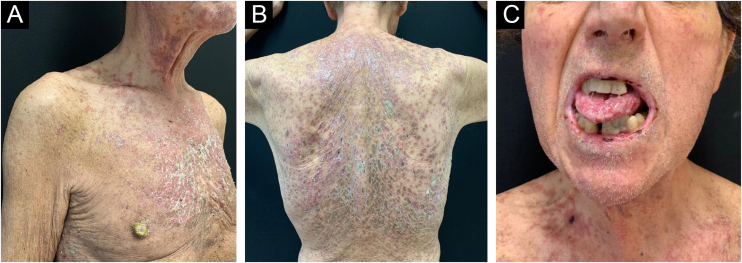
(A and B) Disseminated erythematous brownish hyperkeratotic papules and plaques on the trunk. (C) Erosions on the tongue and labial mucosa covered by hemorrhagic crusts.

The histological examination of the skin biopsy revealed a lichenoid interface dermatitis (Fig. 2). Serum IgG anti-desmoglein 3 and IgG anti-envoplakin antibodies were detected by enzyme-linked immunosorbent assay (ELISA). Direct immunofluorescence study of perilesional skin was negative.

**Fig. 2:**
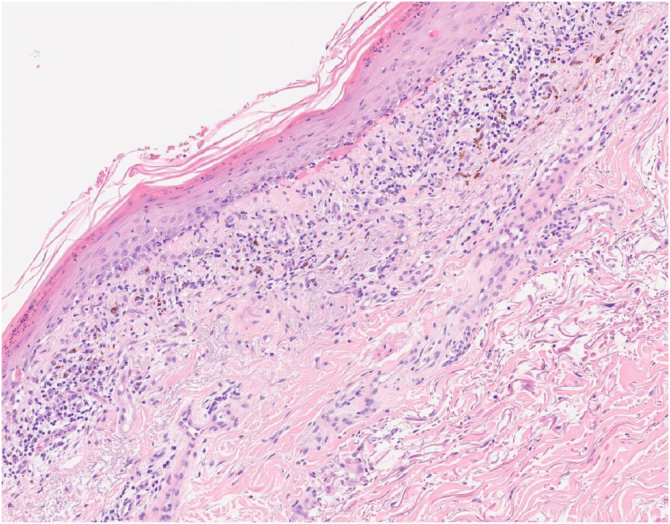
In the dermis, a moderate lymphohistiocytic interstitial inflammatory infiltrate is observed, with moderate pigment incontinence, associated with interface changes (Hematoxylin & eosin; 15×).

Therefore, the diagnosis of paraneoplastic pemphigus was established considering the clinical picture of chronic erosive mucositis associated with a malignant neoplasm, along with the detection of anti-envoplakin antibodies.^2^

In addition to sorafenib for the treatment of hepatocellular carcinoma, the patient was treated with prednisolone 1 mg/kg/day (60 mg) with progressive dose reduction. Approximately six weeks later, there was a significant improvement in the skin lesions (Fig. 3A and B).

Despite treatment with prednisolone and targeted therapy for hepatocellular carcinoma, the symptoms of oral dysphagia and dysphonia worsened due to persistent painful oral erosions, compromising the patient's nutrition. At this point, IGIV (2 g/kg/cycle divided into 5 days) was administered in combination with oral prednisolone 60 mg/day with progressive dose reduction, which resulted in significant clinical improvement. One month after the intravenous cycle, there were only a few lesions on the oral mucosa (Fig. 3C). The patient remained clinically controlled, completing monthly cycles of IVIG and prednisolone 30 mg/day. One year after the diagnosis he died at home following a head injury.

**Fig. 3:**
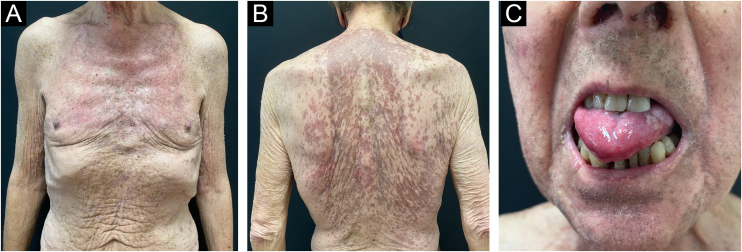
(A and B) Clinical picture six weeks after treatment with systemic corticosteroids with an improvement of skin lesions. (C) Clinical picture four weeks after initial IVIG cycle in association with systemic corticosteroids with significant improvement of oral mucositis.

This case of PNP was associated with a solid tumor. In this context, the evidence supporting the use of rituximab is less clear. Furthermore, the administration of rituximab or other conventional immunosuppressants may promote tumor progression due to significant immunosuppression. Therefore, the combination of systemic corticosteroids with IGIV was considered the safest choice. Additionally, IVIg has been successfully used in other autoimmune blistering diseases such as pemphigus vulgaris and pemphigus foliaceous, being particularly useful in refractory cases to first-line therapy or when the use of conventional immunosuppressants is not safe.^4^, ^5^

In conclusion, our case illustrates a rare association between PNP and hepatocellular carcinoma. Importantly, we highlight the efficacy and safety of IGIV therapy in association with systemic corticosteroids in this condition. To the best of our knowledge, there are only two other cases of PNP related to hepatocellular carcinoma reported in the literature, which were both treated with systemic corticosteroids and azathioprine, with a clinical improvement of the oral lesions in only one.^6^, ^7^

## Authors' contributions

José Ramos: Writing of the manuscript or critical review of important intellectual content; Critical review of the literature; Final approval of the final version of the manuscript.

Cláudia Afonso: Intellectual participation in the propaedeutic and/or therapeutic conduct of the studied cases; Final approval of the final version of the manuscript.

Ângela Roda: Writing of the manuscript or critical review of important intellectual content; Critical review of the literature; Final approval of the final version of the manuscript.

Cristina Fonseca: Intellectual participation in the propaedeutic and/or therapeutic conduct of the studied cases; Final approval of the final version of the manuscript.

## Financial support

None declared.

## Conflicts of interest

None declared.
